# Smokeless Tobacco‐Associated Nitrosamines Impact on Aryl Hydrocarbon Receptor: In Silico and In Vitro Study

**DOI:** 10.1002/jbt.71173

**Published:** 2026-11-07

**Authors:** Mohammad Sajid, Ved Vrat Verma, Varun Rawat, Sonal Srivastava, Pragya Sharma, Mausumi Bharadwaj

**Affiliations:** ^1^ Division of Molecular Genetics and Biochemistry, Molecular Biology Group ICMR‐National Institute of Cancer Prevention and Research Noida Uttar Pradesh India; ^2^ Department of Biotechnology, School of Engineering and Technology Noida International University Greater Noida Uttar Pradesh India; ^3^ School of Biotechnology Gautam Buddha University Greater Noida Uttar Pradesh India; ^4^ Molecular Biology Group ICMR‐National Institute of Cancer Prevention and Research Noida Uttar Pradesh India; ^5^ Academy of Scientific and Innovative Research (AcSIR) Ghaziabad Uttar Pradesh India

**Keywords:** Aryl hydrocarbon receptor, Cytochrome‐P450, smokeless tobacco, tobacco‐specific nitrosamines

## Abstract

Smokeless tobacco (SLT) consumption is a potential health threat around the globe, predominantly in South Asian Countries. SLT products contain several carcinogens, most notably including tobacco‐specific nitrosamines (TSNAs). Aryl hydrocarbon receptor (*AhR*), significantly higher in oral squamous cell carcinoma, is responsible for the recognition of toxic compounds and their elimination by increasing the expression of Cytochrome‐P450 enzymes. However, the impact of TSNAs on *AhR* and *AhR*‐related pathways remains unclear. In this work, we investigate the interaction between *AhR* and various TSNAs present in SLT products by molecular docking and further validate the effects of aqueous extracts (STEs) of these three SLT products on oral cancer cells (CAL‐27) and human embryonic kidney cells (HEK‐293). TSNA molecules docked at the classical 2,3,7,8‐tetrachlorodibenzo‐p‐dioxin (TCDD) binding site of *AhR*. The docking results revealed the comparative binding affinity of all TSNAs molecules against *AhR*. Accordingly, agonist TCDD and NAT showed the highest and lowest binding affinity against *AhR*, respectively. Furthermore, STE exposure led to an increase in cell proliferation at low doses, whereas at high doses, inhibition of proliferation was observed against both cell lines. STE treatment showed an increase in ROS production at low doses, indicating cell transformation. *AhR* and *AhR*‐dependent gene (*CYP1A1*, *CYP1B1*, *Arnt*, and *GST*) expressions were elevated upon exposure to STE. Therefore, induction of the *AhR* signalling network by STE exposure may be pathogenetically involved in oral carcinogenesis and future research on designing an appropriate inhibitor against *AhR* during SLT consumption can assist in the management of oral cancer.

## Graphical abstract

Schematic representation of STE (smokeless tobacco extract) impact on *AhR* and *AhR*‐related genes. TSNAs present in STE induce *AhR* that results in to *AhR* conformational changes and translocation to the nucleus. Inside the nucleus, *AhR* heterodimerizes with Arnt and binds to XRE region of genome. *AhR* = Aryl hydrocarbon receptor; HSP90 = heat shock protein 90; CYP = Cytochrome P450; GST = Glutathione‐S‐transferase; AHRR = Aryl hydrocarbon receptor repressor.

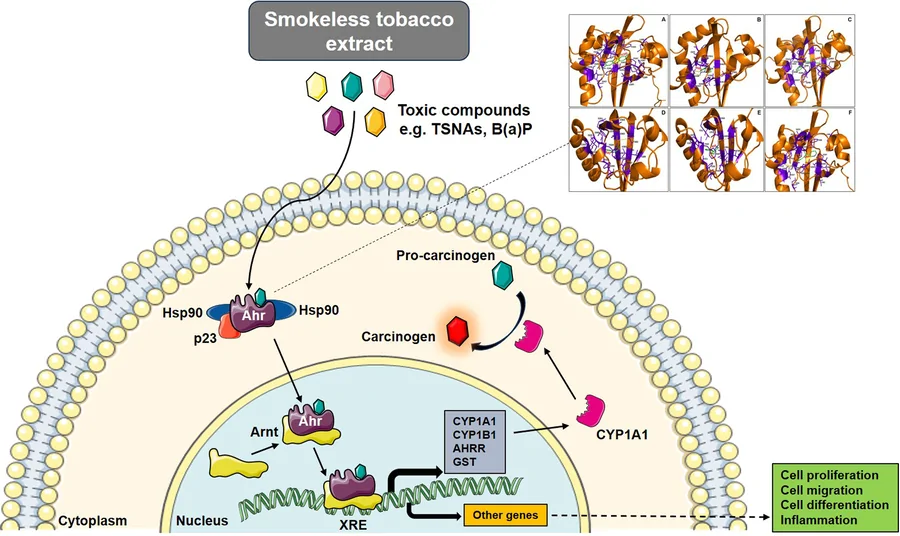

## 1 Introduction

Smokeless tobacco (SLT) products are masticated by more than 300 million people globally and are responsible for more than 0.65 million annual deaths [1]. The consumption of SLT is primarily concentrated in South‐Asian countries. India and Bangladesh alone constitute ~80% of the worldwide SLT user population [2]. In India, adults (21.4%) (Male‐29.6%, and Female‐12.8%) have a habit of chewing SLT products, and it is estimated that approximately 350,000 Indians die each year due to SLT consumption [3]. SLT use is responsible for several forms of cancer (oral, pancreatic, and oesophageal cancers) and is associated with cardiovascular diseases (myocardial infarction and stroke) and poor birth outcomes (low birth weight, untimely birth, lesser brain volumes, and total perinatal mortality) [4, 5, 6].

Aryl hydrocarbon receptor (*AhR*) is a ligand‐driven receptor, a member of the helix‐loop‐helix transcription factor family, which plays a key function in host physiology and several pathophysiologies, including inflammation and cancer [7]. *Ahr* interact with a wide array of ligands that can be categorized into endogenous and exogenous. The endogenous ligands include substances generated by the human intestine and skin microbiota (such as kynurenic acid and indole) and substances created by the human body (such as lipoxin A4 and tryptamine) [7]. The exogenous ligands, xenobiotics (toxic compounds), are sensed by the *AhR*, and activation of *AhR* induces expression of numerous xenobiotic phase I and phase II metabolizing enzymes [8]. After binding of a ligand with *AhR*, *AhR* is transported to the nucleus and heterodimerized with Arnt (Aryl hydrocarbon receptor nuclear translocator). Subsequently, the *AhR*–Arnt heterodimer attaches to definite DNA sequences labelled as dioxin response elements (DREs), which leads to the transcription of target genes [9]. *AhR* modulates the expression of Cytochrome P450 genes (*CYP450*) including *CYP1A1*, *CYP1A2*, *CYP1B1*, and glutathione‐*S*‐transferase (*GST*) [10]. Generally, these enzymes convert toxic compounds into more soluble derivatives to increase their excretion [11]. In contrast to this, xenobiotic metabolizing enzymes can convert inert compounds into hazardous ones [11]. The prolonged exposure of smoke to the mouse corneal endothelium induces *AhR* activation and increased *CYP1B1* expression [12].

SLT products are diverse in their preparative procedures, components, and toxicity. SLT products consist of numerous toxicants and carcinogens, which are responsible for their adverse health effects [13, 14]. The TSNAs are the most effective among 28 identified carcinogens in SLT products [15, 16]. IARC (International Agency for Research on Cancer) grouped two nitrosamines, N'‐nitrosonornicotine (NNN) and N'‐nitrosonornicotine ketone (NNK), into Group‐I human carcinogens and attributed them to cancer‐causing agents in SLT users [17]. STE demonstrated their cytotoxic effects on various cells (both cancer and normal cells) [18, 19, 20]. STE‐treated human oral carcinoma cells (SCC‐25) showed inhibition in cell proliferation, ROS production, and apoptosis induction [19]. Snus (a Swedish SLT) extracts significantly inhibited cell viability, increased ROS formation, and induced apoptosis in human umbilical vein endothelial cells, suggesting the toxicological effects of Snus extracts [20]. A moist smokeless tobacco 1S3 (an American SLT) extract treatment to human monocytic cell line Mono Mac 6 (MM6) causes a significant inhibition in cell viability and an increase in apoptotic cell death [21]. Nicotine, an active component of tobacco products, can encourage the propagation of non‐small cell lung cancer (NSCLC) and human breast cancer cells through activating the nicotinic acetylcholine receptors (nAChRs) [22].

There have been numerous studies performed concerning SLT‐induced cytotoxicity, but the exact molecular mechanism is still ambiguous. Further, *in‐vitro* studies of Indian SLT products on cancer cells are very limited. Therefore, in this study, the interactions of different TSNAs against *AhR* were performed and the effects of three popular STE, on the expression of *AhR* and *AhR*‐dependent gene. We have selected one *Moist‐snuff* and two *Qiwam* products based on our previous observations, where these SLT products contain high levels of nicotine and TSNAs [22, 23]. Further, these SLT products harbour diverse microbiomes with a prevalence of harmful microbes that have metabolic potential that can be involved in TSNAs synthesis by nitrogen metabolism [24, 25]. This study will assist in determining the molecular mechanism involved in SLT‐induced carcinogenesis through modulating the *AhR*‐dependent pathway.

## 2 Materials and Methods

### 2.1 Chemicals

Three SLT products (One *Moist‐snuff* and two *Qiwam*) were procured from retail shops in Noida, India. Tricarboxylic acid (TCA), Sodium bicarbonate, Sulforhodamine B (SRB), and DMEM/F‐12 (Dulbecco's Modified Eagle Medium/Nutrient Mixture F‐12) were acquired from Sigma Aldrich (Sigma‐Aldrich Chemicals Pvt. Ltd., Bangalore, India). 2′,7′‐dichlorodihydrofluorescein diacetate (H_2_DCFDA) were obtained from Thermo Scientific (Waltham, MA). Antibiotic/Antimycotic solution was procured from BioString (BioString, Johnstown, PA). The oral cancer cell line (CAL‐27) was obtained from ATCC, whereas HEK‐293 cells were obtained from the NCCS, Pune, India. The chemicals that were not stated here were also of analytical quality.

### 2.2 Molecular Docking Study of TSNAs Molecules in the Deep Cavity of AhR

The Autodock tool was used for receptor and ligand preparation, whereas the Autodock Vina tool was used to perform molecular docking studies [27]. The three‐dimensional coordinates of *AhR* were searched and retrieved from the PDB database (PDB ID: 7ZUB, Chain D). The small molecule compounds database PubChem was used to download the various forms of TSNAs molecules. The OpenBabel tool was used to convert the file format of ligands (sdf) to an AutoDock‐readable file format (pdbqt). For receptor preparation, heteroatoms were removed, polar hydrogens and Kollmann's charges were added, and the total charge distribution on residues was also checked accordingly. Similarly, ligand structures were prepared by adding appropriate Gasteigner charges and setting the appropriate torsion values. The AutoSite tool was used to find a binding pocket site on the Chain D region (especially around the PAS fold region) of the receptor; the x, y, and z coordinates were noted, and accordingly, the configuration file was made defining the binding pocket dimensions. Now, receptor‐ligand docking was performed using the Autodock Vina platform. Further, the docked complex was visualized using LigPlot (for 2D interactions) and PyMol (for 3D visualization) [27, 28].

### 2.3 ADMET Analysis

For ADMET (absorption, distribution, metabolism, excretion, and toxicity) studies of TSNAs, SMILES format of each TSNA was analyzed using the SwissADME and pkCSM tools [29, 30]. PubChem was used to get SMILES of respective TSNAs from the database, viz. NNN (PubChemID:27919), NNK (PubChemID:47289), NAB (PubChemID:14335), NAT (PubChemID:528366) and NNAL (PubChemID:104856).

### 2.4 Smokeless Tobacco Product Extract (STE) Preparation

With some modifications, the aqueous extract of three SLT products was prepared using Mitchell and colleagues' method [32]. SLT product (4 g) was taken in incomplete DMEM media (40 ml), and the mixture was incubated for 24 h at 37°C in a shaker incubator at 300 rpm. The tubes were centrifuged at 125 × g (10 min), and the supernatant was collected and centrifuged at 14,000 × g for 1 h at 4°C using a refrigerated centrifuge (Sigma‐4K15). Next, the supernatant was passed through a 0.22 μm sterile syringe filter under sterile conditions and stored at −80°C until further use.

### 2.5 Cell Proliferation Assay

The effects of STE on cell proliferation were monitored by the SRB assay as described earlier [33]. The cells were seeded in 96‐well plates (1 × 10^4^ cells/well) and incubated for 24 h at 37°C with 5% CO_2_. STE was applied to the cells (in triplicate) with different concentrations (0.097%, 0.195%, 0.390%, 0.781%, 1.56%, 3.125%, 6.25%, 12.5%, 25%, 50%, 75%, 100%) and kept for 24 h and 48 h. Next, 10% (w/v) TCA (100 µL/well) was supplemented and placed at 4°C for 1 h. The plates were washed with sterile MQ water (4x) and dried at RT. After that, 0.05% (w/v) SRB solution (100 µl) was added and incubated at RT (30 min) and then washed with 1% (v/v) acetic acid (4x) to eliminate the unbound SRB. The plates were kept for drying in a warm environment (30°C). Finally, 10 mM Tris‐base solution (pH 10.5) was used (200 µL/well) to dissolve the bound SRB, and plates were read at 564 nm after shaking for 5 min using a SPECTROstar Nano microplate reader (Biotron Healthcare Pvt. Ltd, Mumbai, India). The percentage of viable cells was calculated by the following formula, where untreated cells was taken as a negative control and media alone was used as a background control to remove the possibility of any contamination.

$$\text{Cell viability}\;\left(\%\right)=\frac{\left(\text{Mean OD of treated cells}-\text{Mean OD of media blank}\right)\times 100}{\left(\text{Mean OD of untreated cells}-\text{Mean OD of media blank}\right)}$$

### 2.6 In Vitro Scratch Assay

In vitro, the cell migration test was accomplished as per the previous protocol defined by Liang et al. [34]. Briefly, CAL‐27 cells (1 × 10^5^ cells/well) were seeded in 12‐well plates and placed in a CO_2_ incubator till a complete monolayer was formed in each well. The scratch was created in each well, the media was discarded, and detached cells were removed with sterile PBS washing. STE treatment was given to cells at different concentrations (0.5%, 2.5%, 1.0%, and 5.0%). At 0 h and 24 h, the scratches were observed under a fluorescence microscope (Radical Scientific Equipment Pvt. Ltd, Ambala, India) and photographed at 10x objective lens magnification. The width of the wounds was determined using Radical ProCam Software. The wound healing (%) was calculated by the following formula.

$$\text{Wound healing}\;(\%)=\frac{[\text{Mean width}\;\left(\text{at}\;0\text{hr}\right)-\text{Mean width}\;\left(\text{at}\;24\text{hr}\right)]\times 100}{\text{Mean width}\;(\text{at}\;0\text{hr})}$$

### 2.7 Intracellular Reactive Oxygen Species (ROS) Detection Assay

The cells were seeded into 12‐well plates and incubated at 37°C with 5% CO_2_ for 24 h or once the cells reached 80%–90% confluency. Next, treatment with three STE was given at concentrations of 0.25%, 0.5%, 1%, 2.5% and 5.0%. After incubation (24 h), the media was removed, and ROS‐detecting dye, H_2_DCFDA (12 µM), was added for 30 min and incubated at 37°C. Next, cells were washed with PBS to remove excess dye. Finally, ROS production was monitored under a fluorescence microscope (Radical Scientific Equipment Pvt. Ltd, Ambala, India) at 20× objective lens magnification with an excitation wavelength of 488 nm and an emission wavelength of 525 nm. The untreated cells were taken as a negative control. Consequently, ROS fluorescence intensities were calculated and expressed directly, rather than normalized to cell viability ratios.

### 2.8 Expression analysis of AhR and AhR‐dependent genes by qRT‐PCR

Whole RNA was isolated employing the TRIzol method [35]. The integrity of isolated RNA was checked on an agarose gel, and concentrations were determined by UV/Vis spectrophotometer (µ2 Microdigital, Nabi, Pangyo, South Korea). cDNA was prepared using the GoScript™ Reverse Transcription System (Promega kit). At first, nuclease‐free water (1 µL), oligo dT (5 µL), random primer (5 µL), and template RNA (1 µL) were taken in a PCR tube and heated at 70°C for 5 min (one‐cycle PCR). Nuclease‐free water (7.3 µL), 5X buffer (4 µL), MgCl_2_ (1.2 µL), nucleotide mix (1 µL), RNAse inhibitor (0.5 µL), and reverse transcriptase (1 µL) were added to the PCR tube from the previous reaction to perform two‐cycle PCR (25°C for 5 min, 42°C for 60 min). The concentration of the prepared cDNA was checked using a UV/Vis Nanospectrophotometer (Microdigital, Nabi, Pangyo, South Korea). The expressions of genes *AhR*, *CYP1A1*, *CYP1B1*, *Arnt*, *GST*, and *GAPDH* (internal control) were evaluated using the specific primer sets (Table S1). For RT‐PCR, the total reaction volume of 10 µL was used, including 1x SYBR master mix (5 µL), 10 pM of each primer (1 µL each), cDNA (100 ng/µL), and nuclease‐free water (2 µL). Thermal cycler settings involved the following steps: UDG activation (50°C for 2 min), initial denaturation (95°C for 2 min), and denaturation (95°C for 15 s), followed by 40 cycles of annealing/extension step (60°C for 1 min).

### 2.9 Statistical Analysis

The data are denoted by mean ± standard error of means (SEM), and statistical significance between the two groups were calculated by a post‐hoc test (Tukey's multiple comparison test) for Ordinary One‐Way ANOVA or Ordinary Two‐Way ANOVA tests (GraphPad Prism v 8.0, San Diego, CA). The results were typically taken into consideration as statistically significant when *p* ≤ 0.05.

## 3 Results

### 3.1 ADMET Properties of Tobacco‐Associated Toxic Compounds

TSNAs exhibited moderate values for absorption (Log P and Log S), which favours membrane permeation without extreme hydrophobicity (Table 1). Higher predicted Caco‐2 permeability likely suggests good oral absorption in‐vitro surrogate. More than half (> 50%) of the respective TSNAs remain unbound in human plasma; this substantial free fraction can be available for target engagement and clearance. Lower clearance rates were observed for NNN and NAB, suggesting longer systemic exposure when compared to other TSNAs (Table 1). Broadly speaking, all TSNAs, naphthalene, benz(a)anthracene, and chrysene showed high LogP values and adequate Caco‐2 permeability, indicating likely cellular uptake. None of the TSNA was predicted to be *CYP450* substrates/inhibitors or hERG inhibitors, which decreases metabolic and cardiac interaction concerns. AMES mutagenicity and carcinogenicity showed increased mutagenicity and strong genotoxicity concern for tobacco‐associated toxic compounds, except naphthalene. None of the tobacco‐associated toxic compounds displayed hepatotoxicity (Table 1).

**Table 1:** ADMET analysis of tobacco associated toxic compounds.

Type		Absorption			Distribution		Metabolism		Excretion		Toxicity			
		Log P	Log S	Caco‐2 permeability	Fraction unbound (human)	BBB	CYP450 substrate profile	CYP450 inhibitor profile	Total clearance	OCT‐2 substrate	AMES mutagenicity	Hepato‐toxicity	Carcino‐genicity	hERG inhibition
TSNAs	NNN	1.96	−1.87	1.33	0.525	0.159 (yes)	No	No	0.392	No	Yes	No	Yes	No
	NAT	2.06	−2.04	1.354	0.504	0.188 (yes)	No	No	0.898	No	Yes	No	Yes	No
	NNAL	2.3	−1.47	1.001	0.602	0.266 (yes)	No	No	0.595	No	Yes	No	Yes	No
	NAB	2.17	−2.16	1.351	0.502	0.178 (yes)	No	No	0.394	No	Yes	No	Yes	No
	NNK	2.02	−1.52	1.277	0.448	0.024 (yes)	No	No	0.929	No	Yes	No	Yes	No
Naphthalene		1.99	−3.45	1.574	0.144	0.434	No	CYP1A2 inhibitor	0.198	No	No	No	No	No
Benz(a)anthracene		2.74	−5.62	1.466	0.174	0.642	CYP3A4 substrate	CYP1A2 inhibitor	0.125	No	Yes	No	Yes	hERG II inhibitor
Chrysene		2.69	−5.66	1.417	0.187	0.658	No	No	−0.824	No	Yes	No	Yes	No
Acetaldehyde		0.8	0.06	1.317	0.529	0.285	No	CYP1A2 inhibitor	0.886	No	Yes	No	Yes	No
Formaldehyde		0.39	−0.81	1.484	0.804	−0.031	No	No	0.544	No	Yes	No	Yes	No
Crotonaldehyde		1.27	−0.56	1.498	0.696	0.045	No	No	0.276	No	Yes	No	Yes	No

Abbreviations: BBB = Blood brain barrier, Caco‐2 = Human colorectal adenocarcinoma cells, CYP450 = Cytochrome P450, hERG = Human ether‐à‐go‐go‐related gene, OCT‐2 = Organic cation transporter‐2, TSNAs = Tobacco specific nitrosamines.

### 3.2 Binding Mode of TSNAs in the Deep Cavity of AhR

The detailed, comprehensive literature survey suggests a lack of information about the molecular mechanism of SLT‐mediated *AhR* modulation and its expression mechanism. Therefore, the molecular docking studies of various TSNAs molecules against *AhR* were performed to analyze the reaction mechanism of *AhR* and the role of the binding affinity of TSNAs molecules in the gene expression of *AhR*. Additionally, a detailed binding interactions analysis of TSNAs molecules in the deep cavity of *AhR* was performed to understand a comparative binding affinity analysis of these molecules against the *AhR* macromolecule. Based on the availability of related literature, we observed that among the known agonists for *AhR*, 2,3,7,8‐tetrachlorodibenzo‐p‐dioxin (TCDD) is one of the most potent molecules that can have the capability to show good binding affinity against the *AhR*. The comparative variability among the binding interactions network of all TNSA molecules, including TCDD, has been better understood by examining all docked complex structures (Figure 1). The molecular docking outcomes of our studies revealed the highest binding affinity for TCDD with docking scores −9.6 kcal/mol and least binding affinity for NNAL with docking scores −6.9 kcal/mol; however, the other TSNAs molecules showed noticeable binding affinity with the docking scores of −7.3 kcal/mol, −7.3 kcal/mol, −7.1 kcal/mol, and −7.0 kcal/mol for NNN, NAB, NAT, and NNK respectively (Table S2). Further, the binding cavity of *AhR*, the molecular docking results of TCDD revealed that there was no hydrogen bonds were formed between TCDD and the functional residues of the *AhR* (Figure 1A). However, the binding interaction network of TCDD in the binding cavity of *AhR* has been stabilized by non‐covalent bonding electrostatic interactions and Van der Waals forces with twenty residues of the binding cavity such as F287, T289, H291, P297, L308, G321, Y322, F324, I325, C333, A334, L335, S336, H337, S347, I349, F351, S365, V381, and Q383 (Figure 1A). The molecule NNN forms two hydrogen bonds (highlighted by yellow dotted lines) one with each C333, and S336 (Figure 1B). Additionally, amino acid residues F295, Y322, F324, I325, I349, F351, S365, A367, and V381 interact by non‐covalent electrostatic interactions and Van der Waals forces with the NNN (Figure 1B). The NNK forms four hydrogen bonds (highlighted by yellow dotted lines) two with residues Q383 and one with each T289 & H291 (Figure 1C). Additionally, amino acid residues F295, G321, Y322, I325, C333, S336, H337, S346, L353, V363, V381, and T382 of *AhR* interacted by non‐covalent electrostatic interactions and Van der Waals forces with the NNN (Figure 1C). Further, we observed that the NAT forms two hydrogen bonds (highlighted by yellow dotted lines) one with each S365 and Q382 (Figure 1D). And amino acid residues T289, H291, F295, P297, G321, F324, I325, C333, S336, I349, F351, L353, V363, A367, V381, and Q383 of *AhR* interacted by non‐covalent electrostatic interactions and Van der Waals forces with the NAT (Figure 1D). Next, we analyzed the binding interactions network of the molecule NAB where we observed that hydrogen bonds (highlighted by yellow dotted lines) with residues L351 and Q383 (Figure 1E). In addition to that we observed that NAB also stabilized by non‐covalent electrostatic interactions and Van der Waals forces with amino acid residues T289, H291, F295, P297, G321, F324, I325, C333, F351, L353, V363, A367, V381, and Q383 (Figure 1E). Further, we observed that NNAL forms four hydrogen bonds (highlighted by yellow dotted lines) two with residues C333 and one with each G321 and S336 (Figure 1F). Other than these NNAL interacted by non‐covalent electrostatic interactions and Van der Waals forces with the residues T289, H291, F295, P297, F308, Q323, F324, I325, H337, S346, I349, F351, L353, V363, A367, V381, T382, and Q383.

**Figure 1:**
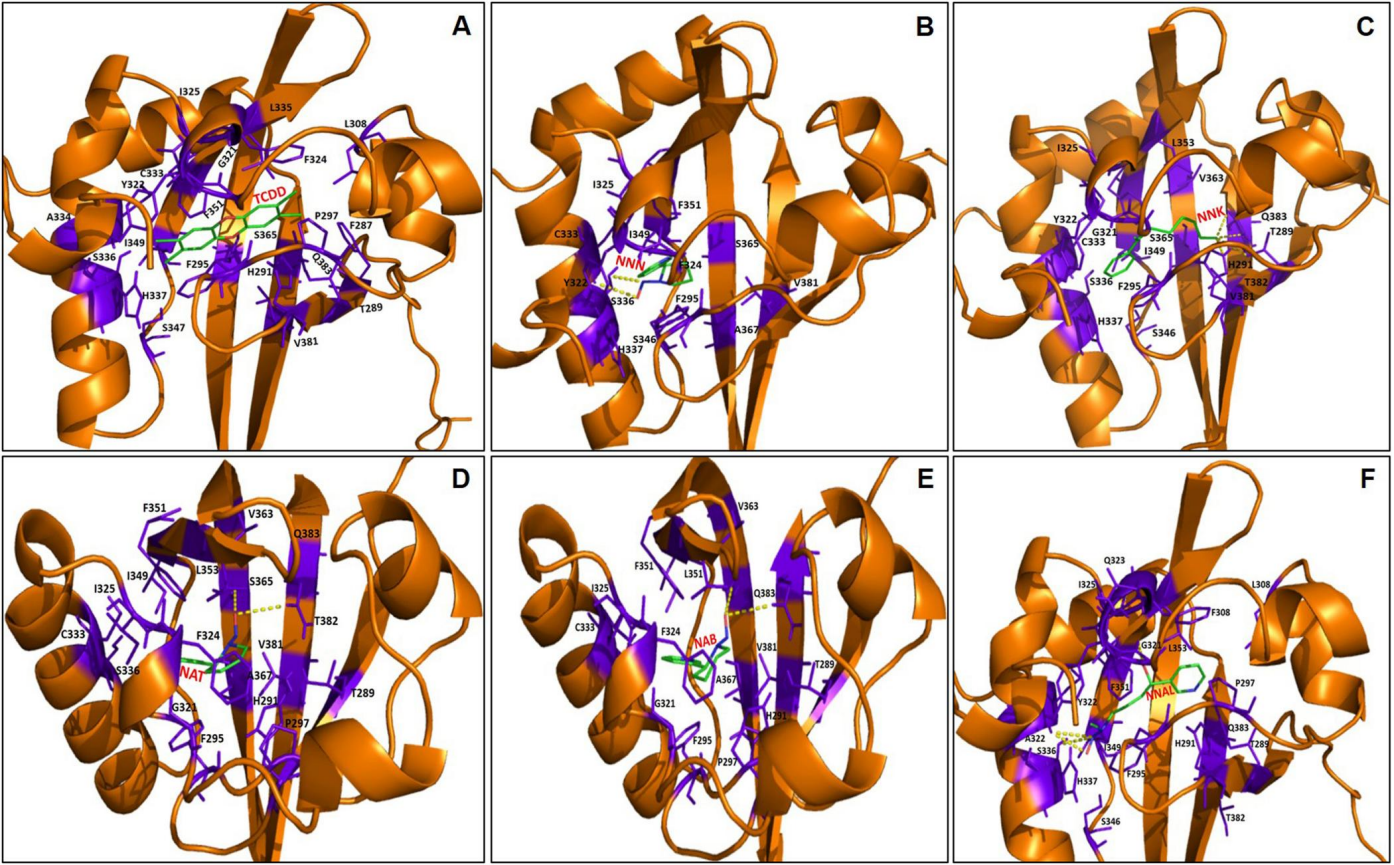
Interactions of different TSNAs in the active site of *AhR*. (A) TCDD interactions with *AhR*; (B) NNN interactions with *AhR*; (C) NNK interactions with *AhR*; (D) NAT interactions with *AhR*; (E) NAB interactions with *AhR*; (F) NNAL interactions with *AhR*. The ligand molecules are shown in green sticks, interacting residues of *AhR* are highlighted in purple sticks, and the protein backbone of *AhR* is represented in orange cartoon. The residues highlighted in purple sticks of *AhR* interact by non‐covalent electrostatic interactions and Van der Wall forces with ligands.

### 3.3 STE Treatment Increases Oral Cancer Cell Proliferation at Low Doses

STE‐mediated impact on cell proliferation was evaluated up to 24 h and 48 h for CAL‐27 and HEK‐293 cells. At 24 h incubation, the SLT‐1 and SLT‐3 showed cytotoxic effects on HEK‐293 cells in a dose‐dependent manner, whereas SLT‐2 displayed inhibition up to 1.56% concentration and proliferation of cells from 3.125% to 25% (Figure 2A). Interestingly, at 48 h incubation, SLT‐2 does not show the significant cytotoxic effect on HEK‐293 cells till 25% of the concentration (Figure 2B). Similarly, at 24/48 h incubation, it was observed that SLT‐2 did not significantly alter the proliferation of CAL‐27 cells upto 25% whereas lower doses (0.195% and 0.39%) of SLT‐1 induce proliferation of CAL‐27 cells (Figure 2C,D).

**Figure 2:**
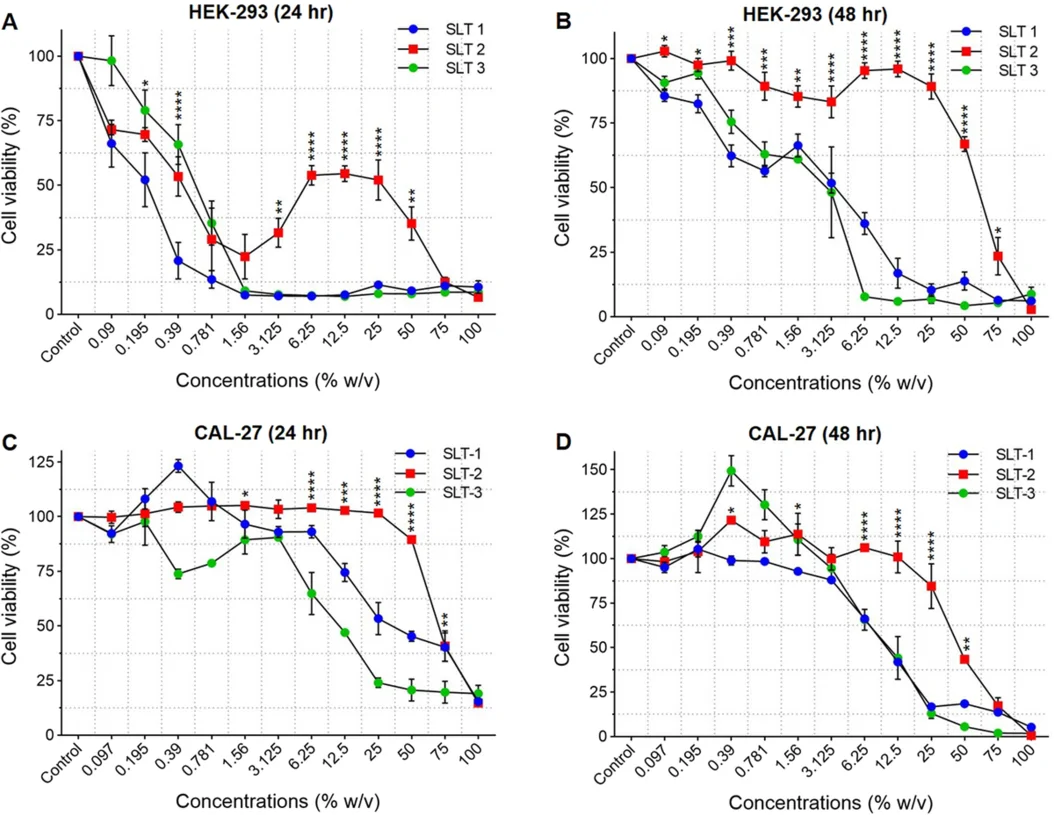
Cytotoxic effect of three STE on HEK‐293 and CAL‐27 cells. (A) 24 h STE treatment on HEK‐293 cells; (B) 48 h STE treatment on HEK‐293 cells; (C) 24 h STE treatment on CAL‐27 cells; (D) 48 h STE treatment on CAL‐27 cells. The cells were treated with different concentrations of SLT aqueous extracts (0.097%, 0.195%, 0.39%, 0.78%, 1.56%, 3.125%, 6.25%, 12.5%, 25%, 50%, 75%, and 100%). The SRB assay was performed to assess cell viability. The experiment was done in triplicate, and the mean ± standard error of measurement was calculated for each data point. An Ordinary Two‐Way ANOVA with Tukey's multiple comparison test was computed among groups. *p < 0.05, **p < 0.01, ***p < 0.001, ****p < 0.0001.

### 3.4 STE Treatment Promoted ROS Production

The effect of STE on ROS production in HEK‐293 and CAL‐27 cells was monitored under the fluorescence microscope. After the HEK‐293 cells were treated with the three diverse STE, the intracellular ROS levels increased as observed by the increase in the intensity of the fluorescence (Figure 3A). However, SLT‐2 also showed significantly increased ROS production at concentrations 0.25%, 2.5%, and 5% treatment when compared with SLT‐1 and SLT‐2 (Figure 3B). At 0.5% concentration, both SLT‐2 and SLT‐3 showed considerably elevated ROS generation than SLT‐1, whereas at 1% concentration SLT‐2 and SLT‐3 showed higher ROS generation as compared to SLT‐1 (Figure 3B). For CAL‐27 cells, SLT‐1 and SLT‐2 induced significant ROS production at concentration 2.5%, while SLT‐2 treatment also showed an increase in ROS generation at a 0.5% concentration as compared SLT‐1 (Figure 4A,B). To understand the STE‐mediated impact on cancer cells, further experiments were performed on oral cancer cells (CAL‐27).

**Figure 3:**
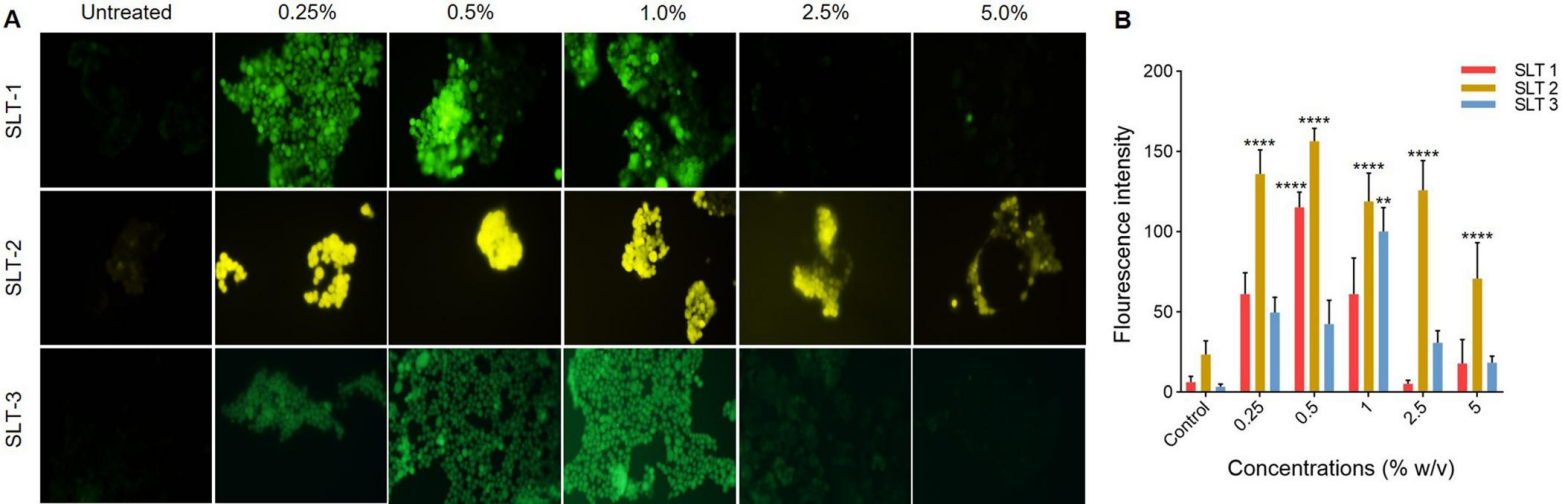
The effect of STE on ROS production inside HEK‐293 cells. (A) The HEK‐293 cells were treated with different concentrations (0.25%, 0.5%, 1%, 2.5%, and 5.0%) of three different STE (SLT‐1, SLT‐2, and SLT‐3) for 24 h. The cells were stained with ROS‐detecting dye (H2DCFDA) and observed under a fluorescence microscope. (B) The fluorescence intensity was calculated using ImageJ software and plotted using the bar graph. An Ordinary Two‐Way ANOVA with Tukey's multiple comparison test was computed among groups. **p < 0.01, ****p < 0.0001.

**Figure 4:**
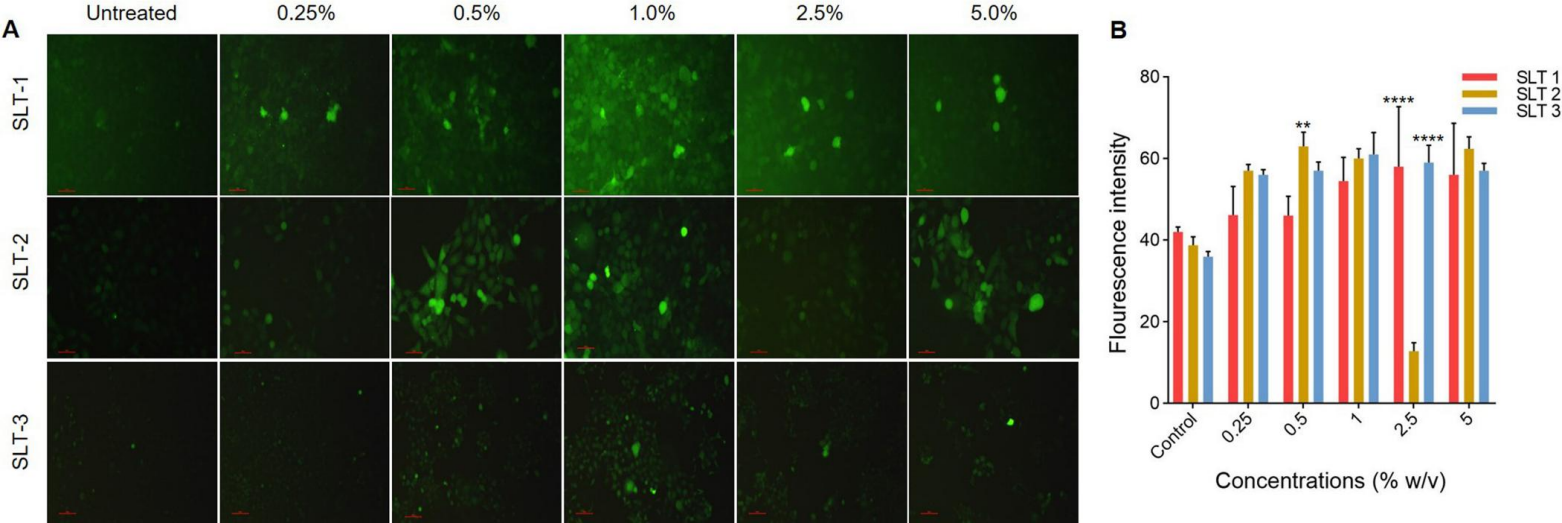
The effect of STE on ROS production inside CAL‐27 cells. (A) The CAL‐27 cells were treated with different concentrations (0.25%, 0.5%, 1%, 2.5%, and 5.0%) of three different STE (SLT‐1, SLT‐2, and SLT‐3) for 24 h. The cells were stained with ROS‐detecting dye (H2DCFDA) and observed under a fluorescence microscope. (B) The fluorescence intensity was calculated using ImageJ software. An Ordinary Two‐Way ANOVA with Tukey's multiple comparison test was computed among groups. **p < 0.01, ****p < 0.0001.

### 3.5 STE Treatment Induces the Movement of Oral Cancer Cells

The scratch assay was executed to monitor the migration property of CAL‐27 upon treatment with STE. After 24 treatments with three STE, it was observed that there was a notable increase in the migration of CAL‐27 cells (Figure 5). SLT‐1 treatment showed a significant increase in the migration of oral cancer cells at concentrations 0.5% and 1% whereas SLT‐2 and SLT‐3 treatments remarkably enhances the movements of oral cancer cells at concentrations 0.25%, 0.5%, 1% and 2.5% as compared to untreated cells (control) (Figure 5). At 5% concentration, SLT‐3 showed significant increase in migration of oral cancer cells than control cells.

**Figure 5:**
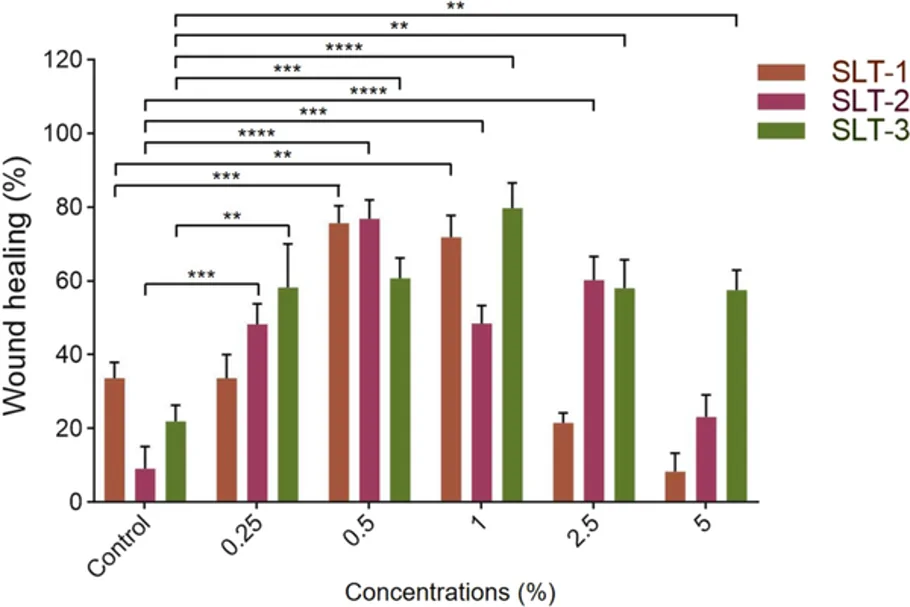
In vitro oral cancer cell (CAL‐27) migration. The CAL‐27 cells were treated with different concentrations (0.25%, 0.5%, 1%, 2.5%, and 5.0%) of three different STE (SLT‐1, SLT‐2, and SLT‐3). The scratch area was captured at 0 h and 24 h and measured by ImageJ software. Data are presented as the mean of three independent experiments ± SEM. An ordinary two‐way ANOVA with Tukey's multiple comparison test was computed among groups. **p < 0.01, ***p < 0.001, ****p < 0.0001.

### 3.6 STE Modulates *AhR* and *AhR*‐Dependent Gene Expression

The expression of *AhR* and *AhR*‐dependent gene expression was monitored upon treatment with two STEs (SLT‐1 and SLT‐2) based on their previous effects on proliferation and ROS generation. The mRNA expression of the *Arnt*, *CYP1A1*, *CYP1B1*, and *GST* treated with SLT‐1 (2.5% concentration) displayed a significant increase as compared to the control (Figure 6A–E). However, 0.5% concentration of SLT‐1 treatment showed significantly diminished expression of *AhR*, *CYP1B1*, and *GST* as compared to the control (Figure 6A,D,E). SLT‐2 treatment to oral cancer cells significantly enhances the expression of *AhR*, *Arnt*, *CYP1A1*, *CYP1B1*, and *GST* at 2.5% concentration compared to the control, whereas 0.5% concentration of SLT‐2 showed significantly reduced *AhR* expression (Figure 6F–J).

**Figure 6:**
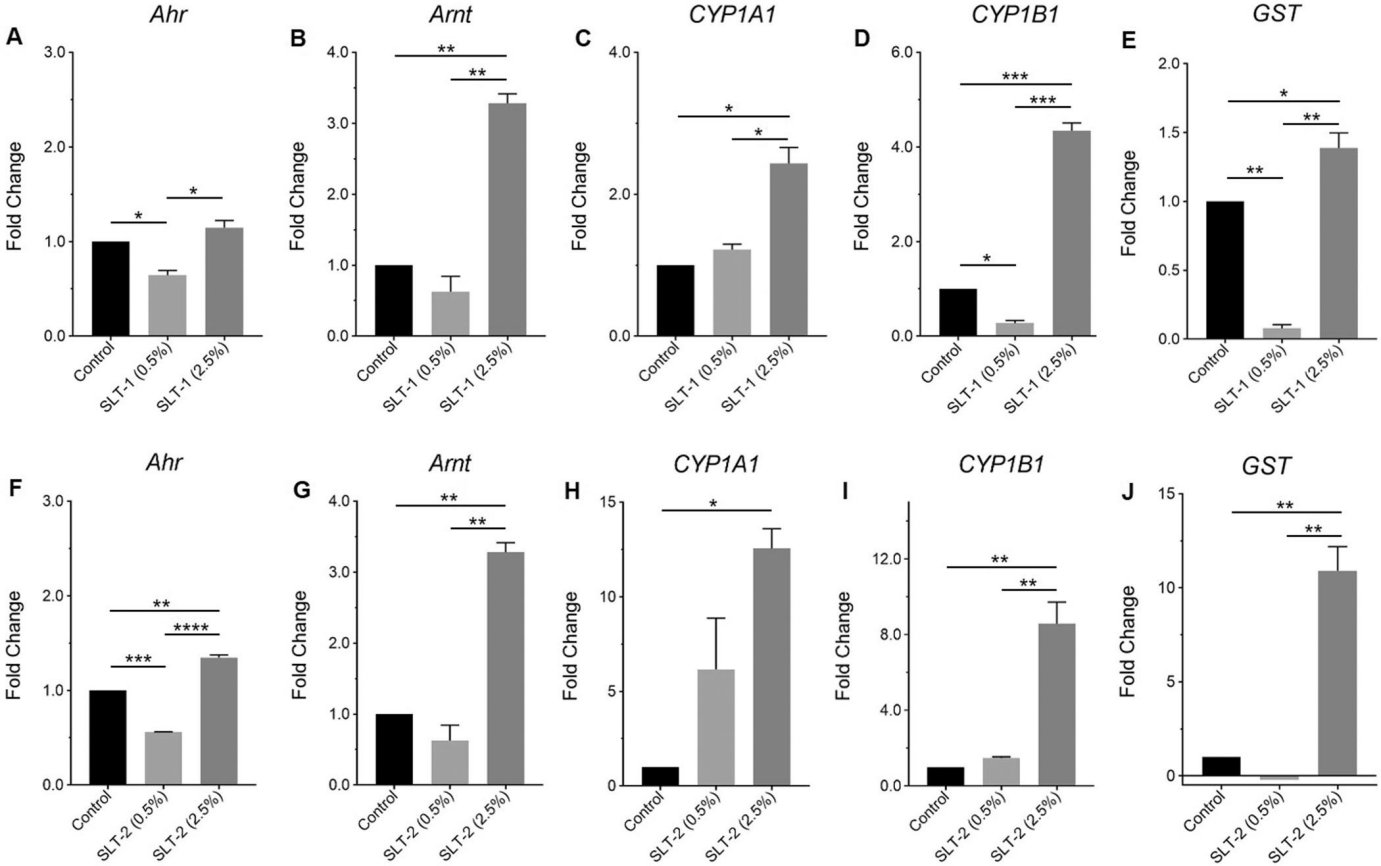
*AhR* and *AhR*‐dependent gene expression after treatment with SLT‐1 and SLT‐2. The relative mRNA expression of genes in CAL‐27 cancer cell lines treated with SLT‐1 extract. (A) *AhR*; (B) *Arnt*; (C) *CYP1A1*; (D) *CYP1B1*; and (E) *GST*. The relative mRNA expression of genes in CAL‐27 cancer cell lines treated with SLT‐2 extract. (F) *AhR*; (G) *Arnt*; (H) *CYP1A1*; (I) *CYP1B1*; and (J) *GST*. Data are presented as the average of three independent experiments ± SEM. An Ordinary One‐Way ANOVA with Tukey's multiple comparison test was applied among groups. *p < 0.05, **p < 0.01, ***p < 0.001.

## 4 Discussion

A huge burden of diseases is caused by SLT consumption worldwide due to the presence of a variety of toxic compounds, many of which may have adverse effects on health, like cancer, cardiovascular, and neurological aberrations [36]. Oral cancer is found to be quite common in people who consume SLT products, as they contain toxic and harmful compounds like TSNAs, Benzo[a]pyrene, and other compounds [37].

*AhR*, an environmental sensor, functions as a transcription factor, with ligand binding to its PAS‐B domain triggering conformational changes that determine its biological response [37, 38, 39]. Further, molecular docking studies in our study revealed that TSNAs showed binding interactions with the *AhR* complex, as evidenced by the results generated from AutoDock Vina and LigPlot. RMSD (root mean square deviation) values of low range (< 3 A°) for true binding pose are considered good, representing good reproduction of the correct pose [41]. Also reported that *AhR* agonists can compete with TCDD in binding to *AhR*, where TCDD demonstrated the highest relative *AhR* ligand binding affinity and the least binding affinity for NNAL. Similar results were shown in our study, where we observed that *AhR* showed good binding affinity with TSNAs. Further, the experimental outcomes of our study showed that STEs played a crucial role in modulating the gene expression of *AhR* and its associated genes. These observations suggest that binding interactions of STE or their carcinogens could influence the gene expression level of *AhR* and *AhR*‐dependent genes. The strength of binding affinity of TSNAs molecules against *AhR* might directly or indirectly affect the gene expression level of *AhR*. In general, based on our docking outcomes, we conclude that TSNAs have comparable good binding affinity towards the *AhR*. Therefore, they will suppress or enhance the gene expression level of *AhR* and its dependent genes, inducing the molecular mechanism related to the promotion of cell proliferation and migration. Additionally, determining ligand binding geometries experimentally and clarifying important chemical changes in the *AhR* signalling cascade are still difficult tasks [42]. Further, before the implementation of key outcomes of our docking part for medical purposes, elaborated experimental validation studies, including an inhibition assay, are needed.

For a particular compound, solubility and permeability are among the key parameters for determining absorption properties [43]. TSNAs showed high hepatotoxicity and carcinogenicity scores, suggesting good metabolic activation to DNA‐alkylating species and association with lung/esophageal/oral cancers in smokers [44]. ADME analysis clearly showed high bioavailability of TSNAs compared with other tobacco‐associated constituents. In addition, the presence of a higher fraction of unbound compound (as observed for TSNAs) may lead to more efficient movement and diffusion across the cell membrane. TSNAs were observed to have carcinogenic potential (as indicated by the Ames toxicity test), which aligns with the previous observations, which showed that the DNA adduct formation was crucial for involvement in tumour‐suppressor genes and other processes [44, 45]. Overall, TSNAs have poor solubility, slow clearance, and high carcinogenic potential showed by ADMET studies, which perfectly align with the fact that IARC classifies NNN and NNK as Group 1 carcinogens [47]. Naphthalene did not show AMES mutagenicity, hepatotoxicity, or carcinogenicity, whereas it exhibited high permeability, suggesting less genotoxic potential despite ubiquitous environmental presence. Further, naphthalene has lower molecular weight and moderate lipophilicity, which allows its rapid absorption via inhalation, ingestion, and dermal contact. Translationally, naphthalene's moderate structure allows it to distribute rapidly into vascularized organs and cleanly cross the blood‐brain barrier, manifesting as acute neurovascular and systemic symptoms [48]. Benz(a)anthracene and Chrysene displayed high log P values, suggesting their high lipophilicity and insoluble properties. Translationally, their oral bioavailability is lower and heavily dependent on lipid vehicles in digestion [49]. However, once absorbed, they exhibit high tissue persistence. They segregate selectively into adipose tissue, mammary glands, and lipid‐rich membranes, leading to long‐term chronic bioaccumulation [49]. Similar to nitrosamines, Benz[a]anthracene and Chrysene showed carcinogenic potential, and a previous study observed that they can promote PAH‐driven squamous cell carcinogenesis via diol‐epoxide adducts [50].

Previously, several studies established that cigarette smoke extract (CSE) can induce the propagation of cancer cells [50, 51, 52]. However, very limited studies regarding STE‐mediated impact on cancer cells were found; therefore, we have selected three commercially sold SLT products that are majorly consumed in India, and their effect was assessed on the oral cancer cell line (CAL‐27) and human embryonic kidney cells (HEK‐293). STE treatment at low concentrations on oral cancer cells (CAL‐27) showed an increase in cell proliferation. Similarly, the CSE from heated tobacco products facilitates the propagation of the human oral squamous cell carcinoma cell line (HSC‐3) at low concentrations (5%) [54]. Our observation on CAL‐27 proliferation (upon STE treatment) is corroborated by a previous study where areca nut (a constituent of SLT products) extract promotes the growth of cancer cells (CAL‐27 cells) [55]. Nicotine, a major constituent of SLT products, showed growth in the proliferation of lung cancer (A549), breast cancer (MCF‐7 and MDA‐MB‐468), and pancreatic cancer (Aspc1) cells through activation of α7‐nAChRs‐mediated signal transduction pathways [22]. At lower doses, the possible mechanism for oral cancer cell proliferation and migration could be due to *AhR* activation, as observed previously, where *AhR* stimulation promotes cellular proliferation and cell migration of cancer cells [55, 56, 57]. Further, it was noticed that high concentrations of STE were found to be toxic to oral cancer cells (CAL‐27) and human embryonic kidney cells (HEK‐293) in a dose‐dependent manner, except SLT‐2, which showed proliferation of HEK‐293 cells within the concentration range of 3.125% to 50%. In a previous report, STE treatment (high doses) resulted in a decrease in the proliferation of human oral squamous carcinoma cells (SCC‐25) [19]. The mechanism involved in the STE‐induced decrease in proliferation at higher doses in these cells could be due to the disruption of the tubulin‐microtubule system (cytoskeleton network) as observed in human lung adenocarcinoma cells (A549) and hepatocellular carcinoma cells (HepG2) upon treatment with STE [18].

The generation of ROS was found to help cancer progression by increasing cell propagation, migration, survival, angiogenesis, and drug resistance [58, 59]. In this study, a significant increase in ROS production was observed in HEK‐293 cells and CAL‐27 cells. CSE from heated tobacco products was found to heighten ROS levels in HSC‐3 cells [54]. Like elevated ROS production in HEK‐293 cells, previous work established a significant increase in the ROS level of various normal cells (human oral keratinocytes, human umbilical vein endothelial cells, Chinese hamster ovary cells, and human oral mucosa fibroblasts) upon treatment with STE [20, 60, 61, 62].

Further, STE treatment increases the migration ability of oral cancer cells (CAL‐27), which is crucial for metastasis. Previously, CSE was found to promote the cell migration of the lung cancer cell lines [64]. Breast cancer cells (MCF‐7) exposed to CSE condensate exhibited increased motility and invasiveness [65]. The mechanism involved in CSE‐induced migration includes induction of EMT (epithelial‐mesenchymal transition), upregulation of pro‐survival pathways, oxidative stress, mitochondrial damage, and the activation of signalling pathways like JAK2/STAT3 [66]. Nicotine can encourage movement and wound healing in NSCLC and breast cancer cells [22]. Due to the chemical composition similarity between STE and CSE condensate (like nicotine, TSNAs, and polycyclic aromatic hydrocarbons), future studies are needed to decipher the role of each toxic molecule in the cancer cell metastasis pathway.

Exogenous toxic compounds are recognized by *AhR* (expressed in all tissues) and metabolized by *AhR* pathway‐related enzymes like *CYP450* [67]. Recent studies revealed that CSE exhibits several agonists for *AhR* and triggers the dioxin signalling pathway [12]. *AhR* was found to be involved in tobacco‐induced tumor progression, and this could be due to a cancer‐promoting hormone, adrenomedullin [68]. TSNAs can be metabolically activated (into more potent carcinogens) by *CYP450* enzymes, mainly *CYP1A1*, which are expressed in oral cells and may initiate carcinogenesis [16]. Interestingly, STE (SLT‐1 and SLT‐2) was found to induce the expression of *AhR*‐dependent enzymes like *CYP450* (*CYP1A1* and *CYP1B1*) and *GST*. The conversion of pro‐carcinogen into carcinogen by *CYP1A1* and *CYP1B1* enzymes is a well‐known mechanism for carcinogenesis [69]. It can be postulated that varying levels of exogenous ligands in a variety of SLT products can induce *AhR* and its related enzymes to different magnitudes. Therefore, it is necessary to comprehensively evaluate the carcinogenic potential of each SLT product concerning its ability to induce *AhR* and its related enzymes.

In this study, crude aqueous extracts of SLT products were used to observe their impact on cell proliferation, migration, free radical production, and *AhR*‐pathway gene expression. To decode the cancer‐promoting mechanism of SLT products, the effects of each toxic compound present in SLT products, like nicotine, TSNAs, polycyclic hydrocarbons, and so forth, need to be studied in vitro. The activation and translocation of *AhR* upon binding with specific carcinogens need to be studied by advanced molecular techniques. Further, in vivo studies are warranted to understand the *AhR*‐mediated carcinogenic mechanism upon regular exposure to SLT products.

## 5 Conclusion

In conclusion, TSNAs present in SLT products showed strong interactions with the *AhR* and low doses of STE induce the proliferation and migration of oral cancer cells and increase oral carcinogenicity by increasing ROS production. STE induces the expression of *AhR* and *AhR*‐dependent enzymes. Therefore, the continuous activation of *AhR* by regular use of SLT products can lead to increased expression of *CYP450* enzymes and more potent carcinogen formation by metabolizing toxic molecules of SLT products. Further, the SLT product's continuous exposure can aggravate the pre‐formed oral cancer cells in SLT users, mediated by *AhR* activation. Hence, future studies are required to confirm the carcinogenic potential of various SLT products through the induction of an *AhR*‐dependent pathway. Further, the advancement of inhibitors that can interrupt the *AhR* signalling pathway might assist in the treatment of SLT‐induced oral cancer.

## Author Contributions

**Mohammad Sajid:** data curation, formal analysis, writing – original draft, conceptualization, methodology, writing – review and editing, visualization. **Ved Vrat Verma:** software, data curation, writing – original draft, formal analysis. **Varun Rawat:** software, data curation, formal analysis, writing – original draft. **Sonal Srivastava:** writing – review and editing. **Pragya Sharma:** writing – review and editing. **Mausumi Bharadwaj:** investigation, validation, supervision, resources, project administration, funding acquisition, writing – review and editing, conceptualization.

## Ethics Statement

The study did not involve clinical studies or patient data.

## Conflicts of Interest

The authors declare no conflicts of interest.

## Supporting information


Supporting File 1

Supporting File 2

Supporting File 3

Supporting File 4

Supporting File 5

## Data Availability

The original contributions presented in the study are included in the article/supplementary material; further inquiries can be directed to the corresponding author.
