# Supplementary material for: Smokeless Tobacco‐Associated Nitrosamines Impact on Aryl Hydrocarbon Receptor: In Silico and In Vitro Study
Source: J Biochem Mol Toxicol. 2026 Nov 7;40(11):e71173. doi: 10.1002/jbt.71173 (PMC13744387; doi:10.1002/jbt.71173)
Supplement: Supplementary file 1 [file JBT-40-e71173-s002.docx]

**Table S1:** Primer sequences used for RT-PCR

| **S. No.** | **Gene** | **Primer** | **Sequence (5'-3')** | **Annealing temperature** |
| --- | --- | --- | --- | --- |
| 1. | *AhR* | Forward | *ACATCACCTACGCCAGTCGC* | 60ºC |
|  |  | Reverse | *TCTATGCCGCTTGGAAGGAT* |  |
| 2. | *CYP1A1* | Forward | *CACCATCCCCCACAGCAC* | 60ºC |
|  |  | Reverse | *ACAAAGACACAACGCCCCTT* |  |
| 3. | *CYP1B1* | Forward | *GCTGCAGTGGCTGCTCCT* | 60ºC |
|  |  | Reverse | *CCCACGACCTGATCCAATTCT* |  |
| 4. | *ArnT* | Forward | *GCTGCTGCCTACCCTAGTCTCA* | 60ºC |
|  |  | Reverse | *GCTGCTCGTGTCTGGAATTGT* |  |
| 5. | *GST* | Forward | *GGGAATCGAAAAAGGAGCAG* | 51ºC |
|  |  | Reverse | *CAGGAGAAAGGAACGAGGAG* |  |
| 6. | *GAPDH* | Forward | *CGAGCCACATCGCTCAGACA* | 60ºC |
|  |  | Reverse | *TAGACGGCAGGTCAGGTCCA* |  |
