# Supplementary material for: Smokeless Tobacco‐Associated Nitrosamines Impact on Aryl Hydrocarbon Receptor: In Silico and In Vitro Study
Source: J Biochem Mol Toxicol. 2026 Nov 7;40(11):e71173. doi: 10.1002/jbt.71173 (PMC13744387; doi:10.1002/jbt.71173)
Supplement: Supplementary file 2 [file JBT-40-e71173-s004.docx]

**Table S2:** Binding affinity and root mean square deviation (RMSD) values of all the binding modes, generated using AutoDock Vina

| **S. No.** | **Ligand** | **Mode** | **Binding affinity (kcal/mol)** | **Distance from the best mode** | |
| --- | --- | --- | --- | --- | --- |
|  |  |  |  | **RMSD lower bound** | **RMSD upper bound** |
| 1. | **NAB**   | 1 | -7.3 | 0.000 | 0.000 |
|  |  | 2 | -7.3 | 0.939 | 1.564 |
|  |  | 3 | -7.2 | 3.191 | 4.260 |
|  |  | 4 | -7.1 | 2.463 | 3.691 |
|  |  | 5 | -7.1 | 2.306 | 3.262 |
|  |  | 6 | -7.1 | 3.841 | 4.789 |
|  |  | 7 | -7.0 | 1.248 | 4.510 |
|  |  | 8 | -7.0 | 2.405 | 3.437 |
|  |  | 9 | -6.9 | 2.065 | 5.245 |
| 2. | **NAT**   | 1 | -7.4 | 0.000 | 0.000 |
|  |  | 2 | -7.4 | 0.767 | 1.286 |
|  |  | 3 | -7.3 | 2.358 | 4.847 |
|  |  | 4 | -7.2 | 1.992 | 4.913 |
|  |  | 5 | -7.2 | 3.010 | 3.888 |
|  |  | 6 | -7.2 | 2.592 | 3.948 |
|  |  | 7 | -7.2 | 1.409 | 4.604 |
|  |  | 8 | -7.1 | 1.457 | 1.944 |
|  |  | 9 | -7.1 | 2.338 | 5.028 |
| 3. | **NNK**   | 1 | -7.1 | 0.000 | 0.000 |
|  |  | 2 | -7.0 | 2.304 | 2.361 |
|  |  | 3 | -6.9 | 2.373 | 5.993 |
|  |  | 4 | -6.9 | 1.443 | 1.979 |
|  |  | 5 | -6.8 | 2.091 | 6.124 |
|  |  | 6 | -6.8 | 2.710 | 5.929 |
|  |  | 7 | -6.8 | 1.834 | 2.558 |
|  |  | 8 | -6.8 | 2.495 | 7.796 |
|  |  | 9 | -6.7 | 2.320 | 6.499 |
| 4. | **NNAL**   | 1 | -7.0 | 0.000 | 0.000 |
|  |  | 2 | -6.9 | 2.299 | 6.679 |
|  |  | 3 | -6.9 | 1.984 | 2.897 |
|  |  | 4 | -6.9 | 1.274 | 1.533 |
|  |  | 5 | -6.8 | 1.991 | 6.447 |
|  |  | 6 | -6.8 | 2.193 | 6.468 |
|  |  | 7 | -6.7 | 2.432 | 6.662 |
|  |  | 8 | -6.7 | 1.836 | 1.924 |
|  |  | 9 | -6.7 | 2.738 | 6.970 |
| 5. | **NNN**   | 1 | -7.4 | 0.000 | 0.000 |
|  |  | 2 | -7.3 | 2.042 | 2.506 |
|  |  | 3 | -7.2 | 2.782 | 4.222 |
|  |  | 4 | -7.0 | 2.656 | 4.433 |
|  |  | 5 | -7.0 | 2.947 | 4.188 |
|  |  | 6 | -7.0 | 6.704 | 8.282 |
|  |  | 7 | -6.9 | 2.222 | 4.835 |
|  |  | 8 | -6.9 | 2.805 | 4.431 |
|  |  | 9 | -6.9 | 2.852 | 4.263 |
| 6. | **TCDD**   | 1 | -10.1 | 0.000 | 0.000 |
|  |  | 2 | -10.1 | 0.048 | 6.332 |
|  |  | 3 | -10.1 | 0.072 | 6.777 |
|  |  | 4 | -10.1 | 0.074 | 2.416 |
|  |  | 5 | -9.6 | 1.072 | 1.342 |
|  |  | 6 | -9.6 | 1.082 | 6.837 |
|  |  | 7 | -9.5 | 1.035 | 2.669 |
|  |  | 8 | -8.9 | 1.748 | 6.656 |
|  |  | 9 | -8.9 | 1.014 | 6.411 |
